# *Amp*C-BETA Lactamases among *Enterobacteriaceae* Isolated at a Tertiary Hospital, South Western Uganda

**DOI:** 10.9734/BBJ/2014/10570

**Published:** 2014-09

**Authors:** Martha Nakaye, Freddie Bwanga, Herbert Itabangi, Iramiot J. Stanley, Mwambi Bashir, Joel Bazira

**Affiliations:** 1 Department of Microbiology, Mbarara University of Science and Technology, P.O Box 1410, Mbarara, Uganda; 2 Department of Microbiology, Makerere College of Health sciences, P.O 7072 Kampala, Uganda

**Keywords:** Enterobacteriaceae, AmpC beta lactamase, antibiotic, resistant

## Abstract

**Aim:**

To characterize *AmpC*-beta lactamases among *Enterobacteriaceae* isolates from clinical samples at Mbarara Regional Referral Hospital.

**Study Design:**

Laboratory-based descriptive cross-sectional study

**Place and Duration of Study:**

Microbiology Department, Mbarara Regional Referral Hospital and MBN clinical Laboratories, between May to September 2013.

**Methodology:**

This study included 293 *Enterobacteriaceae* isolates recovered from clinical specimens that included blood, urine, stool and aspirates. *Amp*C Beta lactamase production was determined using disc placement method for cefoxitin at a break point of <18mm. Common *AmpC* plasmid mediated genes were *EBC, ACC, FOX, DHA, CIT* and MOX were; was determined by Multiplex PCR as described by Hanson and Perez-Perez.

**Results:**

Plasmid mediated *Amp*C phenotype was confirmed in 107 of the 293 (36.5%) cefoxitin resistant isolates with 30 isolates having more than one gene coding for resistance. The commonest source that harbored *Amp*C beta lactamases was urine and *E. coli* was the most common *Amp*C producer (59.5%). The genotypes detected in this study, included EBC (n=36), FOX (n=18), ACC (n=11), CIT (n=10), DHA (n=07) and MOX (n=1).

**Conclusion:**

Our findings showed that prevalence of *Amp*C beta-lactamase at MRRH was high (39.6), with EBC as the commonest genotype among *Enterobacteriaceae* Urine and *E. coli* were the commonest source and organism respectively that harbored *AmpC* beta-lactamases. There‘s rational antimicrobial therapy and antibiotic susceptibility tests should be requested by health workers especially patients presenting with urinary tract infections and bacteraemias.

## 1. INTRODUCTION

*Enterobacteriaceae* are common causes of hospital and community acquired infections. The main stay treatment of these infections is the use of antibiotics, mainly beta-lactam agents, which are the most commonly administered drugs in most resource-poor settings [1].

A key challenge in this treatment has been the tendency for these enteric bacteria to acquire plasmid genetic elements bearing genes for drug resistance. These genes encode for drug resistant proteins (beta lactamase) which have increasingly rendered beta lactam agents less useful in the treatment of the above stated infections [2,3]

Plasmid-mediated *AmpC* beta-lactamases have risen through the transfer of chromosomal genes for the inducible *AmpC* beta-lactamase onto plasmids, this transfer has resulted in plasmid-mediated *AmpC* beta-lactamases in isolates of *E. coli, Klebsiella pneumoniae, Salmonella* species, and *Proteus mirabilis* [4,5]

*AmpC* beta-lactamases which are often plasmid mediated hydrolyze all β-lactam antibiotics except cefepime and carbapenems and confer resistance to cephalothin, cefazolin, cefoxitin, most penicillins and beta-lactam inhibitor combinations (broad multidrug resistance) continue to be a major problem in health care settings[6]. Although published literature has evidence that levels of antibiotic-resistant bacteria are high and continue to rise elsewhere in Africa [7,8].

There’s insufficient information about occurrence and detection of *AmpC* at Mbarara Regional Referral Hospital. Knowledge of *AmpC* beta-lactamase occurrence is essential to guide the clinicians towards the appropriate anti-microbial treatment [9]. A serious challenge facing clinical laboratories is that clinically relevant *AmpC*-mediated resistance is not always detectable in routine susceptibility tests. This study evaluated presence of *AmpC*-beta lactamases among *Enterobacteriaceae* isolates from clinical samples at Mbarara Regional Referral Hospital.

## 2. MATERIALS AND METHODS

### 2.1 Study Design

This was a Laboratory based descriptive cross sectional study conducted between May to September 2013 at Mbarara Regional Referral Hospital microbiology laboratory and MBN Clinical Laboratories Kampala, Uganda.

### 2.2 Study Samples

These included Non-repetitive Gram negative isolates (*Enterobacteriaceae*) obtained from various clinical samples that were received in the Microbiology Laboratory were sub cultured on MacConkey agar and incubated at 35–37°C for 16–24 hours. In house made Triple sugar iron agar, urease, oxidase, indole, motility and citrate test were used for biochemical identifications as published by [10].

### 2.3 Laboratory Detection of *Amp*C Beta Lactamases

#### 2.3.1 Disc diffusion test

This was performed by the Novel disc displacement method. In the center of the Muller-Hinton agar plate, imipenem (10μg) (Inducer) disc was applied. At the distance of 20 mm, the disc of cefotaxime (30μg) was placed. From this disc, in a circular manner, clockwise, the discs of cefoxitin (30μg) (Inducer), ceftriaxone (30μg), ceftazidime (30μg), ceftazidime + clavulanic acid (30/10μg), and aztreonam (30μg) were placed such that any two adjacent discs were 20mm apart from center to center. On overnight aerobic incubation at 37°C, the diameters of zones of inhibition were measured and interpreted according to Nagdeo et al., 2012 [11]. A break point <18mm zone diameter for cefoxitin was taken as resistant to cefoxitin, no increase of zone size with addition of inhibitor (ceftazidime-clavulanic acid) and flattening zone of inhibition for cefotaxime (30μg), ceftazime (30μg), ceftriazone (30μg), aztreonam (30μg), ceftazidime-clavulanic acid (30/10μg) towards imipenem (10μg) was interpreted as phenotypically positive for *AmpC.*

#### 2.3.2 Genotypic characterization

All isolates were screened for the resistance genes MOX and DHA, EBC and FOX, ACC and CIT by a multiplex PCR assay using universal primers [12–14]. Criteria for multiplexing was Based on molecular weight (base pairs) and melting temperatures, and Primers were paired as follows; MOX and DHA, EBC and FOX, ACC and CIT. Total DNA targeting both genomic and plasmid DNA of the *Enterobacteriacae* was extracted by the boiling method as published by Perez-Perez and Hanson [15]. All PCR amplicons were verified by gel electrophoresis.

### 2.4 Quality Control

For phenotypic detection, Known AmpC producers or Indicator strains (*E. coli* ATCC 25922 and *E. coli* ATCC 35218 were cultured along the test organisms as negative and positive controls respectively and their zone diameters measured and interpreted according to CLSI guidelines. For genotypic detection, Negative controls were PCR reagent mixtures with the addition of sterile nuclease free PCR water in place of template DNA and positive controls were*: Escherichia Coli* CCUG 58543 and *Escherichia Coli* CCUG 62975.

### 2.5 Data Analysis

Data was entered in Microsoft Excel cleaned and imported to Stata version 11 (Stata Corporation, College Station, TX, USA) statistical packages for analysis. The prevalence of different AmpC Beta lactamase producing organisms and genotypes like MOX, DHA, EBC, ACC, FOX, and ACC obtained after characterization was determined using univariate analysis and cross tabulations.

## 3. RESULTS AND DISCUSSION

**Fig. 1:**
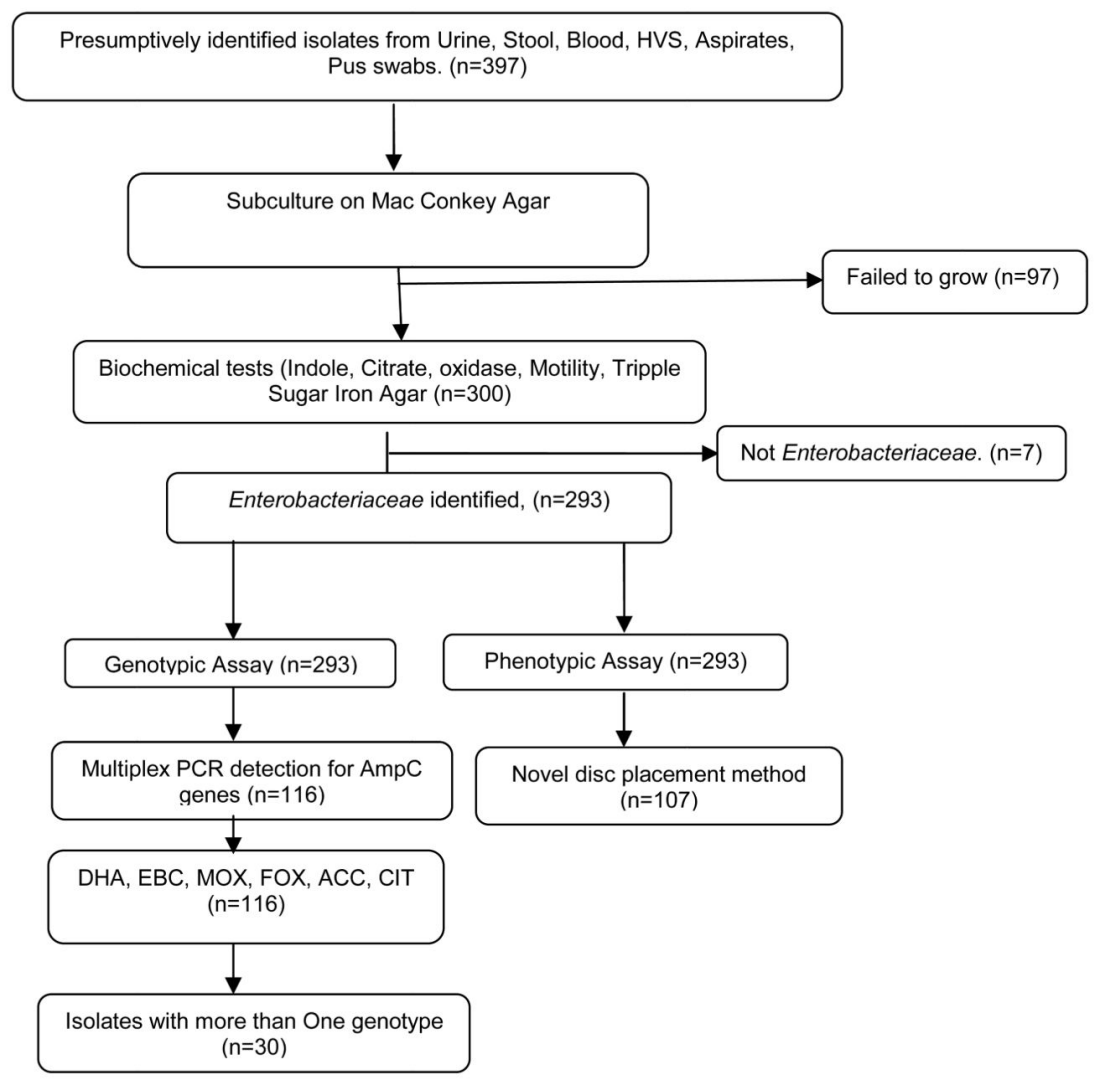
Showing the study profile

The study included 397 clinical isolates sent to the microbiology laboratory for culture and sensitivity collected from different sources, 293 out of 397 clinical isolates were clearly identified as *Enterobacteriaceae* according to our biochemical tests tested by disc diffusion method using Cefoxitin, 107/293 (36.5%) were identified as *AmpC* producers. Multiplex PCR identified 116/293 (39.6%) as *AmpC* producers, with 30 possessing more than one of the following genotypes; DHA, MOX, EBC, ACC, CIT and FOX as shown in Fig. 1

**Fig. 2:**
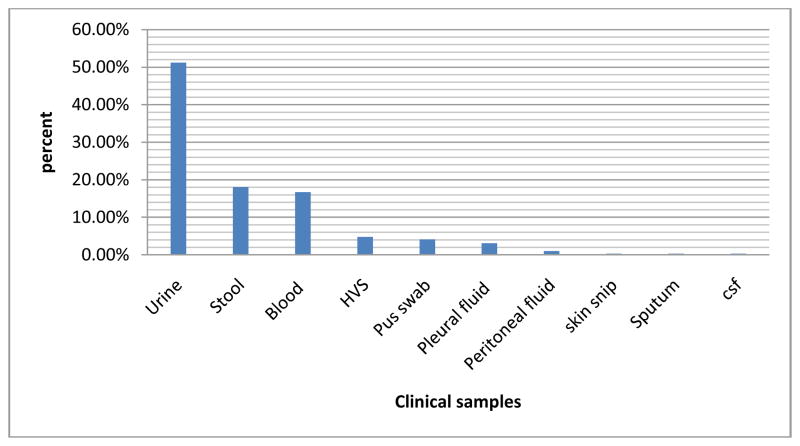
Showing clinical specimens from which study isolates were obtained

Two hundred ninety three *enterobacteriaceae* isolates were obtained and analysed from the following sources and the majority of the isolates were isolated from urine (51.19%) and blood (16.72%) as shown below in Fig. 2.

**Fig. 3:**
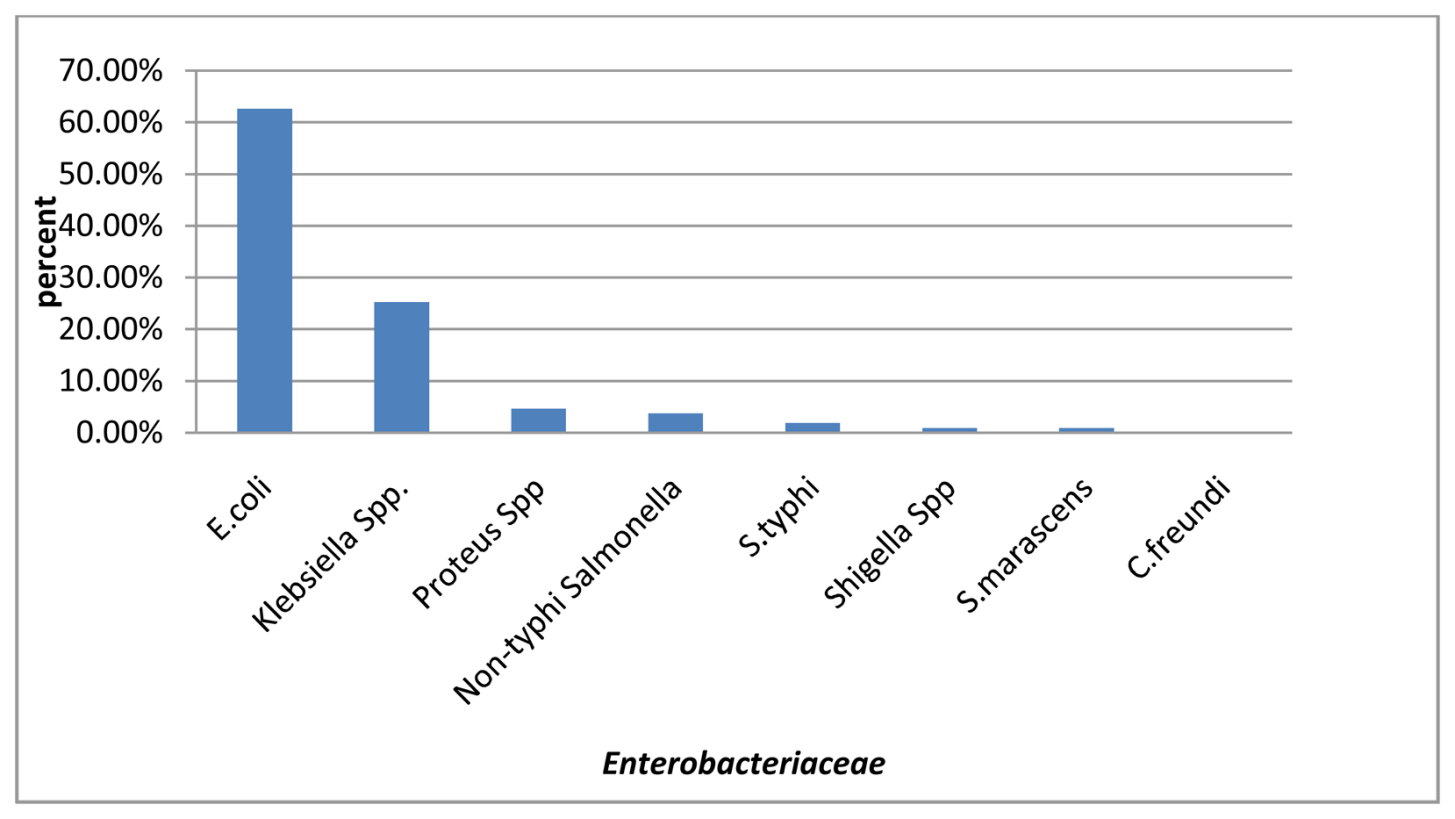
Showing common AmpC producers by phenotypic detection

The overall phenotypic prevalence was 36.52%. Out of 107 *AmpC* producing *Enterobacteriaceae* isolates, detected phenotypically majorly were *E. coli* 67(62.62%)*, Klebsiella Spp.* 27, (25.23%), and *Proteus Spp.* 5(4.67%). *Citrobacter freundii* was a non *AmpC* producer (Fig. 3).

### 3.1 Prevalence of *Amp*C Beta Lactamases by Genotypic Assay

Based on the PCR assays, the overall prevalence by genotypic detection was 39.6% (116/293) *Enterobacteriaceae* bacteria isolates positive for one or more of the *Amp*C beta lactamase resistance genes and the most frequent AmpC gene was EBC followed by FOX (See Table 1).

**Fig. 4:**
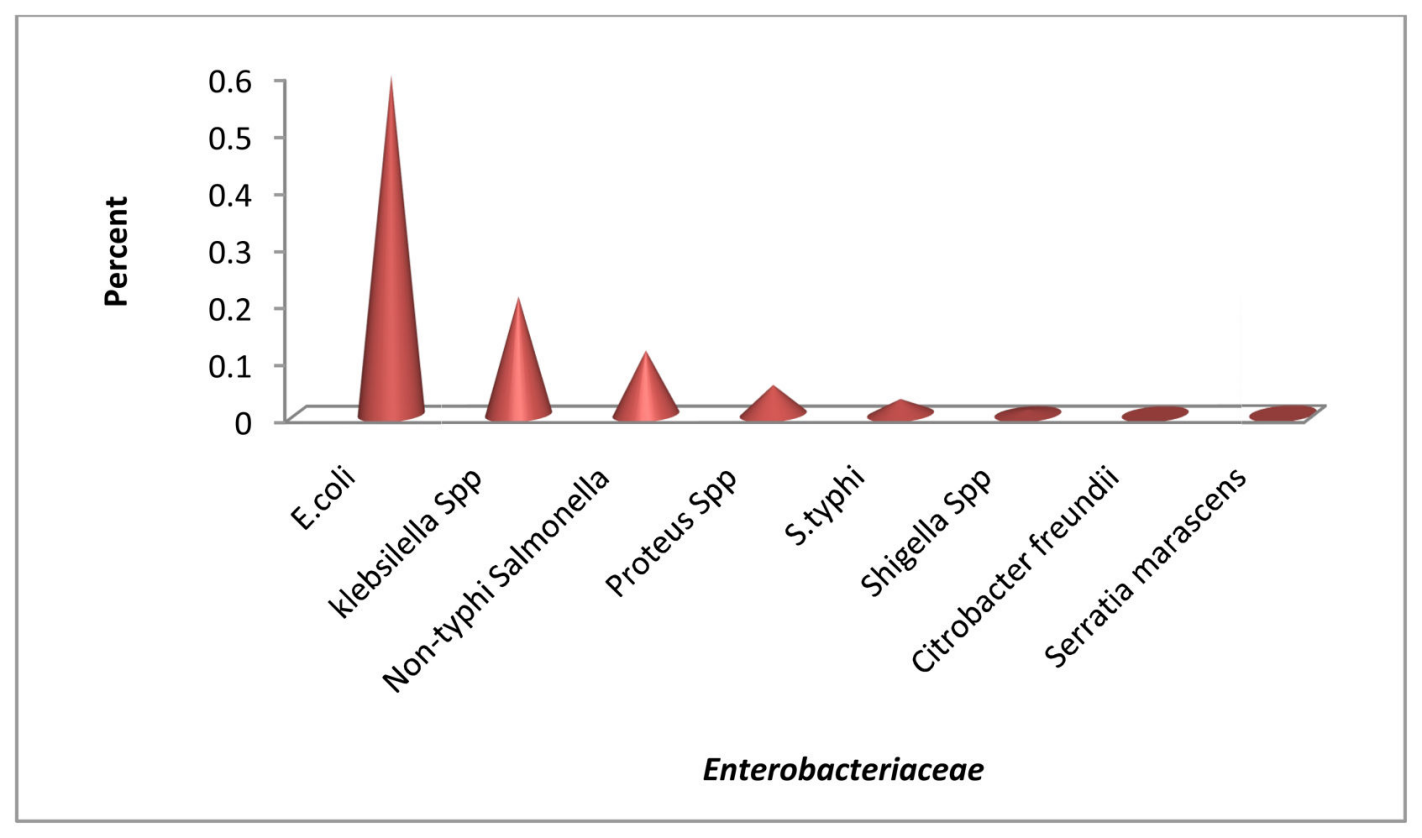
*Enterobacteriaceae* found to harbor *Amp*C beta lactamase genes

Out of 116 isolates that were genotypically positive, *E. coli* (59.48%) possessed most *AmpC* Beta lactamase resistance genes followed by *Klebsiella* Spp. (20.69%) and *Non typhi salmonella* (11.21%) as shown in Fig. 4.

Thirty *Enterobacteriaceae* isolates had multiple *AmpC* resistance genes or more than one gene coding for resistance as shown below (Table 2).

The major *Amp*C beta lactamase genes found in *E. coli* isolates were FOX, followed by ACC and CIT as shown above (Table 3).

Out of 76 isolates with EBC 39(51.3%) were *E. coli,* 18 (23.7%) *Klebsiella* Spp., and 12 (15.8%) were *non-typhi Salmonella* and less prevalent in the rest of the isolates.

Out of 30 isolates with FOX, 21(70.0%) were *E. coli*, 5 (16.7%) *Klebsiella Spp,* 3(10%) *non-typhi Salmonella* and less prevalent in other isolates.

Out of 17 isolates with CIT, 10(58.8%) were *E. coli*, 4 (23.5%) *Klebsiella* Spp*,* 1(5.88%) each of *S. typhi, non-typhi Salmonella* and *Proteus* Spp. The rest of the isolates had no CIT genes.

Out of 21 isolates with ACC, 13(61.9%) were *E. coli*, 6 (28.6%) *Klebsiella* Spp*,* 1(4.8%) each of *non-typhi Salmonella* and *Proteus Spp.* The rest of the isolates had no ACC genes.

Only one MOX gene was found in a *Klebsiella* Spp. Out of the 9 isolates with DHA 4(44.4%) were *Klebsiella* Spp., 3(33.3%) were *E. coli,* and each of *1(11.11%) S. typhi and Proteus Spp*. (Table 3).

The commonest specimen that harbored *AmpC* beta lactamases was urine (61.99%) possessing mainly EBC, FOX and ACC genes. Sputum, skin snips, peritoneal and cerebral spinal fluids didn’t harbor any *AmpC* genes (Table 4).

## 4. DISCUSSION

*AmpC* beta-lactamases mediate resistance to cephalothin, cefazolin, cefoxitin, most penicillins and beta-lactam/beta*-*lactam inhibitor combinations and their over expression confers resistance to broad-spectrum cephalosporins including cefotaxime, ceftazidime, and ceftriaxone [16]

We detected high prevalence (37.19%) of *AmpC* producers among *Enterobacteriaceae* isolates. The findings in this study are similar to a study carried out in India by Anand et al. [16] found the prevalence of phenotypic *AmpC* producers among *Enterobacteriaceae* strains (36.5%).

This prevalence is also higher than 10% *AmpC* prevalence reported by [17] from Kenya [18] and 12% *Amp*C prevalence reported by [19] in Brazil This can be explained that only *E. coli* isolates were studied as opposed to our study that included a number of other species of *Enterobacteriacae*. The other reason for the difference in our findings could be the different methods used., that included, combined disc diffusion, Tridimensional and Hodge test) The prevalence is higher because the genotypic method used in this study is more sensitive compared to the above methods.

Majority of the *AmpC* genes containing *Enterobacteriacae* were in urine and blood. This is consistent with the studies done by [16,19]. This implies the rational use of antibiotics especially patients with UTIs and bactermias, therefore carbapenems should be considered during the patient management.

In the study, sizeable numbers of cefoxitin resistant isolates were not positive for *AmpC* production by the disc placement method or multiplex PCR; this warrants further investigation into the other mechanisms of resistance and their laboratory detection.

Thirty clinical isolates expressed more than one plasmid-mediated *AmpC* beta-lactamases. Two reasons could explain this observation. First, the inability of current phenotypic tests to accurately detect the type of transferable *AmpC* beta-lactamase does not allow for the differentiation of multiple AmpC enzymes. Second, it is possible that there is a limit to the amount of *Amp*C β-lactamase that a bacterial cell can accommodate and still be a viable pathogen according to [16]. A single type of test (PCR) will not be able to accurately characterize the resistance mechanisms in these complex organisms.

## 5. CONCLUSION

Overall, prevalence of *AmpC* beta-lactamases was high (39.6%). The commonest genotype detected was EBC (n= 76) and FOX (n=30) and the least detected genotype was MOX (n=1). Thirty *Enterobacteriaceae* had more than one genotype. The common AmpC producers in this study were *E. coli, (59.48%)* followed by *Klebsiella* Spp*.,(20.69%) and Non-typhi Salmonella (11.21%)* and the commonest source or specimen that harbored *Amp*C producers was urine (47.4%). The genotypic detection was better than the phenotypic detection.

## Figures and Tables

**Table 1:** Prevalence of *Amp*C beta lactamase genes among *Enterobacteriaceae*

*Amp*C beta lactamase resistance gene	Frequency (n=293)	Percentage (%)
FOX	30	10.24
EBC	76	25.94
CIT	17	5.8
ACC	21	7.17
DHA	9	3.07
MOX	01	0.34

**Table 2:** *Enterobacteriaceae* with more than one gene

*Bacteria species*	No. of *Amp*C resistance genes per isolate				
*S. typhi*	**One**	**Two**	**Three**	**Four**	**Total**
*S. marcescens*	2	1	0	0	3
*C. freundii*	0	0	0	0	0
*E. coli*	0	0	0	0	0
*Klebsiella* Spp	57	8	3	1	69
*Proteus* Spp	12	10	2	0	24
*Non-typhi Salmonella*	5	0	1	0	6
*Shigella*	9	4	0	0	13
Total No. of isolates	1	0	0	0	1
*S. typhi*	86	23	6	1	116

**Table 3:** Distribution of different *Amp*C beta lactamases among *Enterobacteriaceae*

	EBC (%)	FOX (%)	DHA (%)	MOX (%)	CIT (%)	ACC (%)
*Klebsiella* spp (n=52)	18(23.68)	5(16.67)	4(44.44)	1(100)	4(23.53)	6(28.57)
*E. coli* (n=194)	39(51.32)	21(70.00)	3(33.33)	0(0)	10(58.80)	13(61.90)
*S. typhi* (n=5)	2(2.63)	0 (0)	1(11.11)	0(0)	1(5.88)	0(0)
Non-typhi *Salmonella* (n=23)	12(15.79)	3 (10.00)	0(0)	0(0)	1(5.88)	1(4.76)
*C. freundii* (n=2)	0(0)	0 (0)	0(0)	0(0)	0(0)	0(0)
*Proteus* Spp (n=14)	4(5.26)	1(3.33)	1(11.11)	0(0)	1(5.88)	1(4.76)
S. marcescens (n=1)	0(0)	0 (0)	0(0)	0(0)	0(0)	0(0)
*Shigella* (n=2)	1(1.32)	0 (0)	0(0)	0(0)	0(0)	0(0)
Total	76 (100)	30(100)	9(100)	1(100)	17(100)	21(100)

**Table 4:** Distribution of AmpC Beta lactamases in different clinical specimens

Specimens	EBC	FOX	DHA	MOX	CIT	ACC
Urine (n=150)	38	15	4	1	9	10
Blood (n=49)	17	7	0	0	6	3
Puss wab (n=12)	7	1	0	0	01	0
Pleural fluid (n=9)	03	01	0	0	0	02
HVS (n=3)	0	0	0	0	0	01
